# Individualized Anesthetic Management of a Patient With Pheochromocytoma and Concurrent Breast Cancer: A Case Report

**DOI:** 10.7759/cureus.59751

**Published:** 2024-05-06

**Authors:** Ana S Cruz, Rita P Sa, Rui Torres, José P Abreu

**Affiliations:** 1 Anesthesiology, Hospital de Braga, Braga, PRT

**Keywords:** anesthetic challenge, surgical procedure, pheochromocytoma, neurofibromatosis type 1, breast cancer

## Abstract

Pheochromocytomas are rare tumors that present a challenge for surgical and anesthetic management due to their ability to produce significant amounts of catecholamines. This case report highlights the successful management of a 49-year-old woman simultaneously diagnosed with neurofibromatosis type 1, pheochromocytoma, and breast cancer. A key decision by the multidisciplinary team involving endocrinology, general surgery, senology, intensive care, and anesthesiology was to prioritize breast cancer surgery over pheochromocytoma resection. This decision considered the potential for improved prognosis and the need to minimize chemotherapy dosage. The case emphasizes the importance of thorough perioperative preparation, including assessing end-organ damage and optimizing medical therapy. Intraoperative management effectively navigated periods prone to catecholamine release, and postoperative care was closely monitored. This case demonstrates that with meticulous planning, a multidisciplinary approach, and a precise anesthetic strategy, safe anesthesia is achievable for patients with pheochromocytoma undergoing major elective surgeries other than pheochromocytoma resection, adding valuable knowledge to a scarcely documented clinical area.

## Introduction

Pheochromocytomas are rare tumors originating from chromaffin cells in the adrenal medulla. These tumors can produce and release large amounts of catecholamines - adrenaline and noradrenaline. This overproduction can lead to severe hypertension and other symptoms such as headache, sweating, palpitations, and anxiety. The curative treatment for pheochromocytomas is surgical resection [1,2]. The surgical removal of a pheochromocytoma presents significant risks and challenges, particularly for the anesthesiologist. Complications during and after the procedure can include a life-threatening hypertensive crisis, severe hypotension, arrhythmias, and tachycardia. Therefore, such surgeries should involve specialized multidisciplinary teams in centers with considerable expertise in handling these complex cases, involving endocrinology, general surgery, intensive care, and anesthesiology [2,3].

While pheochromocytomas generally occur sporadically, they can also be part of other genetic disorders. These include multiple endocrine neoplasia types 2a and 2b, von Hippel-Lindau syndrome, and neurofibromatosis type 1 (NF1). NF1 is characterized by several clinical features, such as café-au-lait spots, axillary and inguinal freckling, Lisch nodules (iris hamartomas), and neurofibromas. Research indicates that women under 50 with NF1 have a higher risk of developing breast cancer and face increased mortality associated with it [4,5].

We present a case involving a patient with NF1 who was diagnosed with both pheochromocytoma and breast cancer almost simultaneously. In this case, the multidisciplinary team decided to prioritize breast cancer surgery, which was performed before the pheochromocytoma resection. We will describe the perioperative and anesthetic management strategies employed in this complex case.

## Case presentation

Our patient was a 49-year-old woman with a history of hypertension since age 38 without etiological study, dyslipidemia, and smoking. She had no previous surgeries or reported allergies.

**Figure 1:**
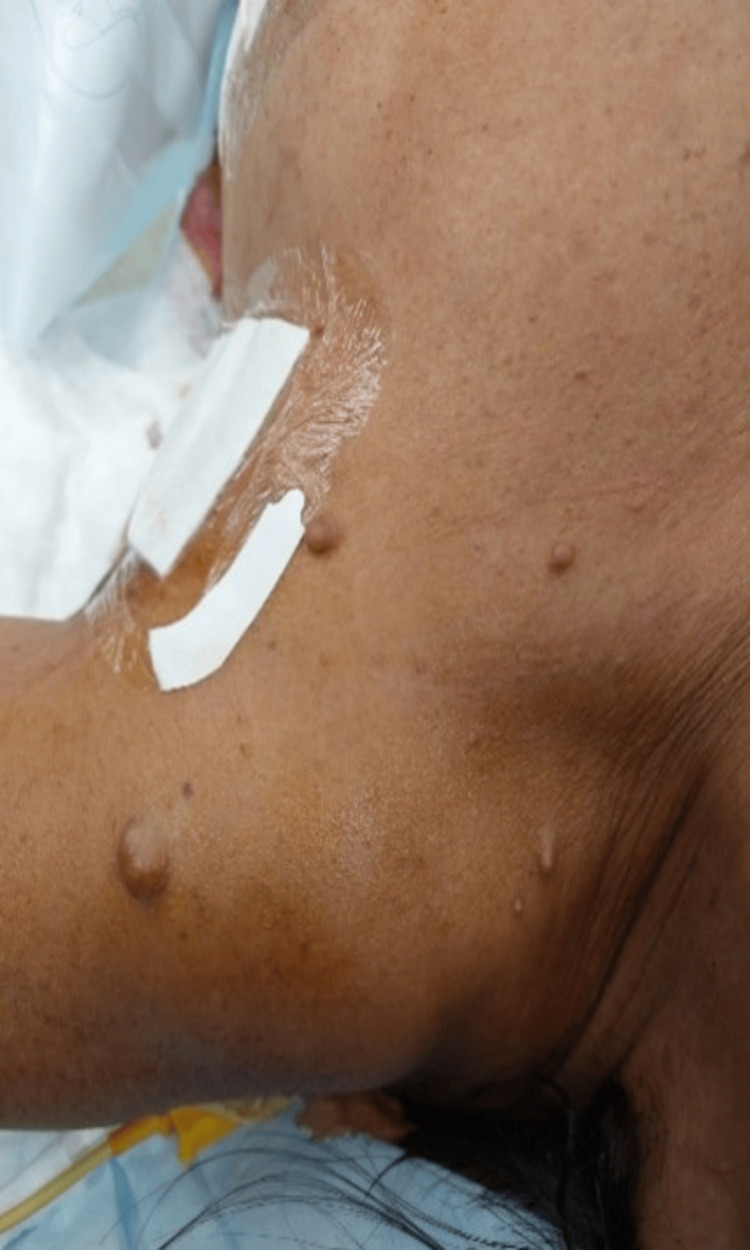
Neurofibromatosis type 1 manifestations - café-au-lait spots and neurofibromas This image depicts the clinical features (café-au-lait spots and neurofibromas) characteristic of neurofibromatosis type 1 (NF1).

In February 2022, she was admitted to the hospital with chest pain, tachycardia, and severe headaches. An electrocardiogram (EKG) revealed an elevation of the ST segment in aVL and diffuse ST depression. The echocardiogram revealed hypokinesia of all middle segments of the left ventricle and moderate depression of the global left ventricular function, with an ejection fraction of 45%. Due to clinical suspicion, total urinary metanephrines were requested. She was diagnosed with catecholamine-induced inverted Takotsubo-like cardiomyopathy. The presence of café-au-lait spots and neurofibromas (Figure 1) indicated that the most likely diagnosis was neurofibromatosis type 1. Later, the diagnosis was confirmed through genetic testing (pathogenic variant c.2041C>T p(Arg681*)).

**Figure 2:**
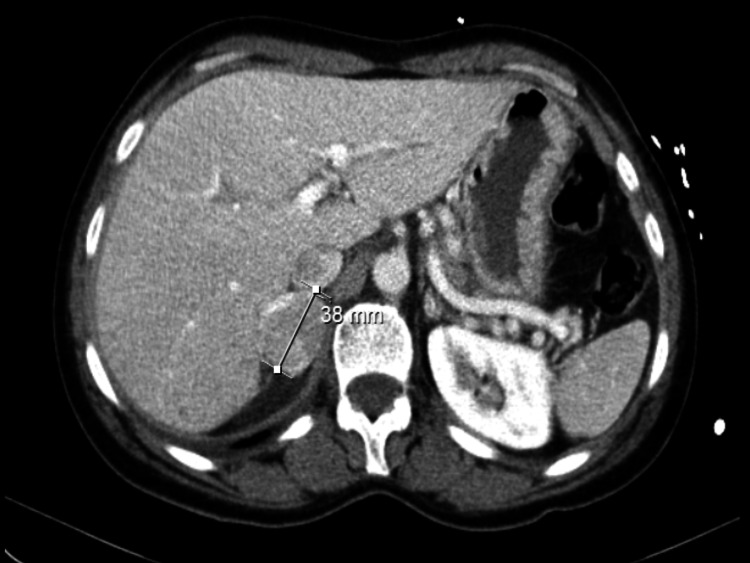
Abdominal CT scan revealing a right adrenal nodule suggestive of pheochromocytoma This CT scan shows the right adrenal nodule, which played a crucial role in diagnosing the patient's pheochromocytoma CT, computed tomography

An abdominal computed tomography (CT) scan identified a right adrenal nodule (Figure 2), and further tests, including urinary and serum catecholamine measurements, confirmed the diagnosis of pheochromocytoma. The patient was referred to Endocrinology and started treatment with an alpha-blocker (phenoxybenzamine, 10 mg thrice daily) initially, then she started a beta-blocker (propranolol, 10 mg thrice daily) and an angiotensin-converting enzyme inhibitor (ramipril, 1.25 mg daily), for blood pressure and heart rate control and control clinical symptoms.

Meanwhile, completely accidentally, the patient self-detected a right breast nodule. This clinical finding was investigated, and she was submitted to breast ultrasound, mammography, biopsy, and breast magnetic resonance imaging (MRI). The study showed a grade 2 invasive and infiltrative triple-positive carcinoma, probably requiring anthracycline-based chemotherapy, hormone therapy, and surgery.

During the staging of breast cancer, an abdominal CT scan revealed a nodule in the opposite adrenal gland, raising the suspicion of bilateral pheochromocytoma and requiring a positron emission tomography-CT scan with 18F-DOPA (18F-fluorodihydroxyphenylalanine) for confirmation.

Facing two simultaneous diagnostics, the team had to prioritize treatments. Given the aggressive nature but the early diagnosis of breast cancer, the patient’s age, and the pending confirmation of bilateral pheochromocytoma, the team prioritized breast cancer surgery to balance anesthetic risks with cancer progression risks. The team decided to proceed with a right breast tumorectomy and sentinel lymph node biopsy, and anesthetic considerations mirrored those for pheochromocytoma surgery.

She was evaluated in pre-operative by the anesthetic team. She reported no complaints of palpitations, diaphoresis, or any other paroxistical symptoms. She was American Society of Anesthesiologists (ASA) Physical Status 4. The airway assessment - Mallampati 2, upper lip bite test 1, absence of limitations in mouth opening or cervical mobility. She showed stable blood pressure (maximum systolic 145 mmHg, diastolic 74 mmHg) and heart rate, normal weight (55 kg), and height (165 cm). She repeated the echocardiogram, which established no cardiac abnormalities (ejection fraction 68%). Routine blood tests and chest X-rays were unremarkable. Postoperative surveillance was ensured in a level 2 unit.

The day before surgery, she was admitted to our hospital and evaluated by endocrinology. The blood pressure and heart rate were monitored, with hemodynamic stability. She received 1 L of glucose-free polyelectrolyte intravenous fluid - "*Freeflax ionolyte*" (Fresenius Kabi Pharma, Portugal) for 12 hours (84 mL/h) and drank clear liquids up to two hours before surgery. She discontinued the alpha-blocker 12 hours before surgery.

In the operating room, the patient was monitored according to ASA standards, including invasive arterial pressure monitoring - maximum systolic 132 mmHg and diastolic 71 mmHg and heart rate of 64 bpm. Additionally, a bispectral index (BIS) electrode and a train of four (TOF) monitors were connected to access anesthetic depth and neuromuscular block, respectively. Pre-medication included 2 mg of midazolam. Hemodynamic stability was a priority, managed with phenylephrine for hypotension and sodium nitroprusside and esmolol for hypertension, among other emergency drugs.

A gentle induction was performed with 100 mcg of fentanyl, 50 mg of lidocaine, 130 mg of propofol, and 30 mg of rocuronium bromide, and the airway was secured with a supraglottic device, iGel mask number 4. General anesthesia was then maintained with sevoflurane. After induction, a right serratus anterior plane block was performed under ultrasound guidance, using 20 mL of 0.375% ropivacaine.

The patient experienced two episodes of hypotension, with a blood pressure reading of 75/40 mmHg. In response, two bolus of 100 mcg of phenylephrine were administered, resulting in recovery to the initial pattern of blood pressure. Due to these episodes, analgesia was administered with caution, comprising 1 g of paracetamol, 100 mg of tramadol, and 30 mg of ketorolac. To prevent postoperative nausea and vomiting, 4 mg of dexamethasone and 4 mg of ondansetron were administered.

The neuromuscular block was reversed by the administration of 100 mg of sugammadex. She regained consciousness without experiencing pain or agitation.

The surgery lasted 91 minutes, with a negative sentinel node biopsy.

Postoperatively, she was monitored in the postanesthetic care unit for eight hours, followed by observation in a level 2 unit care for 48 hours, without any complications. After evaluation by endocrinology, she started her usual medications, such as alpha-blockers, and she was subsequently discharged home five days after surgery.

## Discussion

Successful surgeries in patients with pheochromocytoma undergoing procedures other than pheochromocytoma resection are critically important to report. This is especially true when such cases are associated with syndromes necessitating prior oncological surgery. Our case demonstrates that these surgeries can be conducted safely.

In this case, breast cancer surgery was prioritized over the pheochromocytoma resection due to the potential improvement in prognosis through early tumor excision. This approach also aimed to reduce the need for higher doses of chemotherapy agents, such as anthracyclines, which might adversely affect cardiac function. Reducing anthracycline dosage was intended to preserve the patient's health for future surgeries, including pheochromocytoma resection.

Preoperative preparation is vital for successful perioperative management. The patient was effectively managed preoperatively, guided by endocrinology. This preparation involved assessing end organ damage due to elevated catecholamines, optimizing preoperative medical therapy, confirming the effectiveness of this therapy (such as controlling hypertension and normalizing intravascular volume), and ensuring the patient's readiness for surgery. An alpha-blockade is initiated as the initial step, with the addition of beta-blockade for patients experiencing tachycardia or arrhythmias. It is imperative to refrain from initiating beta-adrenergic blockers prior to alpha-blockers, as the blockade of vasodilatory peripheral beta-adrenergic receptors in the absence of alpha-adrenergic inhibition can result in a further increase in blood pressure. Given the rarity of pheochromocytoma, most knowledge on anesthetic management and perioperative outcomes in patients with concurrent neoplasms comes from small case series [3,4].

The surgery was scheduled after achieving clinical stability, marked by normotension and the absence of adrenergic symptoms. Anesthesia evaluation was timely, ensuring the patient was optimized for a safe procedure. She was admitted a day before surgery, evaluated by endocrinology, and received adequate intravascular filling.

Critical periods were anticipated and well managed intraoperatively, including moments that could trigger catecholamine release, such as airway management, surgical incision, and emergence from anesthesia [1-4]. We chose a supraglottic device to reduce the potential catecholaminergic response associated with laryngoscopy, with a potential hypertensive crisis. But it was ensured that, if necessary, the video laryngoscope would be available. We believe the peripheral nerve block was instrumental in this surgery's success, as it provided effective, prolonged pain control, and reduced the need for intravenous drugs with potential systemic effects, in the post-operative period.

We choose to manage hypotension with phenylephrine, a pure alpha agonist with a short half-life, titrated to achieve the desired effect. The intraoperative hypotensive episodes might have resulted from the late discontinuation of alpha-blockers. Literature suggests stopping alpha-blockers 12 to 48 hours before surgery due to their prolonged effect, but we recommend discontinuing these drugs 24 hours before surgery [3,4].

Close surveillance in the postoperative period is crucial in these situations [1-4].

## Conclusions

To our knowledge, this is a rare case, documenting breast surgery performed on a patient with pheochromocytoma. This case underscores that with thorough perioperative preparation, meticulous anesthetic planning, proper monitoring, cautious induction and emergence, availability of essential medications, effective analgesia, and adequate postoperative surveillance, it is feasible to safely anesthetize patients with pheochromocytoma for elective surgeries beyond pheochromocytoma resection. The success largely stems from the presence of a multidisciplinary team, including endocrinology, general surgery, senology, intensive care, and anesthesiology.

## References

[1] Berends AM, Kerstens MN, Lenders JW, Timmers HJ (2020). Approach to the patient: perioperative management of the patient with pheochromocytoma or sympathetic paraganglioma. J Clin Endocrinol Metab.

[2] Connor D, Boumphrey S (2015). Perioperative care of phaeochromocytoma. BJA Education.

[3] Ramakrishna H (2015). Pheochromocytoma resection. Current concepts in anesthetic management. J Anaesthesiol Clin Pharmacol.

[4] Wiseman D, Lakis ME, Nilubol N (2019). Precision surgery for pheochromocytomas and paragangliomas. Horm Metab Res.

[5] Cimino PJ, Gutmann DH (2018). Neurofibromatosis type 1. Handb Clin Neurol.

